# Improving the diagnostic performance of inexperienced readers for thyroid nodules through digital self-learning and artificial intelligence assistance

**DOI:** 10.3389/fendo.2024.1372397

**Published:** 2024-07-02

**Authors:** Si Eun Lee, Hye Jung Kim, Hae Kyoung Jung, Jin Hyang Jung, Jae-Han Jeon, Jin Hee Lee, Hanpyo Hong, Eun Jung Lee, Daham Kim, Jin Young Kwak

**Affiliations:** ^1^ Department of Radiology, Yongin Severance Hospital, College of Medicine, Yonsei University, Yongin-si, Republic of Korea; ^2^ Department of Radiology, Kyungpook National University Chilgok Hospital, Daegu, Republic of Korea; ^3^ Department of Radiology, CHA University Bundang Medical Center, Seongnam-si, Republic of Korea; ^4^ Department of Surgery, Kyungpook National University Chilgok Hospital, Daegu, Republic of Korea; ^5^ Department of Endocrinology, Kyungpook National University Chilgok Hospital, Daegu, Republic of Korea; ^6^ Department of Radiology, Keimyung University Dongsan Hospital, Daegu, Republic of Korea; ^7^ Department of Computational Science and Engineering, Yonsei University, Seoul, Republic of Korea; ^8^ Department of Endocrinology, College of Medicine, Yonsei University, Seoul, Republic of Korea; ^9^ Department of Radiology, College of Medicine, Yonsei University, Seoul, Republic of Korea

**Keywords:** thyroid cancer, artificial intelligence, ultrasound, learning, digital learning

## Abstract

**Background:**

Data-driven digital learning could improve the diagnostic performance of novice students for thyroid nodules.

**Objective:**

To evaluate the efficacy of digital self-learning and artificial intelligence-based computer-assisted diagnosis (AI-CAD) for inexperienced readers to diagnose thyroid nodules.

**Methods:**

Between February and August 2023, a total of 26 readers (less than 1 year of experience in thyroid US from various departments) from 6 hospitals participated in this study. Readers completed an online learning session comprising 3,000 thyroid nodules annotated as benign or malignant independently. They were asked to assess a test set consisting of 120 thyroid nodules with known surgical pathology before and after a learning session. Then, they referred to AI-CAD and made their final decisions on the thyroid nodules. Diagnostic performances before and after self-training and with AI-CAD assistance were evaluated and compared between radiology residents and readers from different specialties.

**Results:**

AUC (area under the receiver operating characteristic curve) improved after the self-learning session, and it improved further after radiologists referred to AI-CAD (0.679 vs 0.713 vs 0.758, p<0.05). Although the 18 radiology residents showed improved AUC (0.7 to 0.743, p=0.016) and accuracy (69.9% to 74.2%, p=0.013) after self-learning, the readers from other departments did not. With AI-CAD assistance, sensitivity (radiology 70.3% to 74.9%, others 67.9% to 82.3%, all p<0.05) and accuracy (radiology 74.2% to 77.1%, others 64.4% to 72.8%, all p <0.05) improved in all readers.

**Conclusion:**

While AI-CAD assistance helps improve the diagnostic performance of all inexperienced readers for thyroid nodules, self-learning was only effective for radiology residents with more background knowledge of ultrasonography.

**Clinical Impact:**

Online self-learning, along with AI-CAD assistance, can effectively enhance the diagnostic performance of radiology residents in thyroid cancer.

## Highlights

- **Key-finding:** Online self-learning with 3,000 cases improved the diagnostic performance of 26 inexperienced readers (0.679 vs 0.713, p=0.027). Results from an artificial intelligence-based computer-assisted diagnosis program improved it even more (0.713 vs 0.758, p=0.001)
- **Importance:** Online self-learning can improve the diagnostic performance of inexperienced readers from variable backgrounds, and performance can be further enhanced with artificial intelligence-based computer-assisted diagnosis software.

## Introduction

The primary tool for diagnosing thyroid cancer is ultrasonography (US) (1–5). While US exhibits a high diagnostic accuracy, it is inherently operator-dependent and this necessitates appropriate training of related personnel to maintain the quality of examinations. Traditionally, US training isconducted through textbooks, lectures, or one-on-one education sessions between an educator and trainee. While the latter method has been effective, it also has notable disadvantages, such as putting a significant burden on educators and resources and an inability to guarantee a consistent quality of education (6).

Considerable experience is required to make accurate diagnoses with US, and the skill of examiners is known to correlate with the number of scans they have performed (7, 8). Thus, trainees need sufficient practice before performing examinations on people; not only is foundational knowledge of scan techniques or anatomy required but also preparation for actual “diagnosis” or “decision-making” is required. The diagnostic performance of inexperienced readers is known to improve through one-on-one training or structured training in the radiology department (9–11). Considering the pattern-based diagnosis of thyroid nodules in US, simple training with a large number of image examples combined with answers can be helpful when learning how to differentiate benign and malignant thyroid nodules. In a past study, deep learning software achieved similar diagnostic performance to expert radiologists based on 13,560 images (12), and in another, meaningful improvements in diagnostic performance were also observed in college students who had no previous experience in thyroid US, who went through learning sessions using a large training input of image-pathology sets (13).

With the development and commercialization of artificial intelligence-based computer-assisted diagnosis (AI-CAD) in thyroid imaging, potential improvements have been reported in diagnostic performance, particularly among readers with relatively limited experience (14–16). Thyroid Imaging Reporting and Data System (TI-RADS) is commonly used in the evaluation of thyroid nodules, and one study showed that an AI algorithm trained on TI-RADS characteristics outperformed another trained solely on distinguishing benign from malignant nodules (17). Furthermore, another study reported that an AI-proposed new TI-RADS criteria demonstrated superior specificity compared to the established American College of Radiology (ACR) TI-RADS (18). This underscores the potential of AI to enhance diagnostic protocols by leveraging structured reporting systems like TI-RADS. These advancements in AI-CAD not only support diagnostic precision but also provide crucial feedback during the learning phase, directly assisting beginner radiologists. We hypothesize that AI assistance can further aid beginner radiologists in diagnosing thyroid nodules after they undergo a self-learning process, ensuring more consistent and reliable diagnostic outcomes.

In this study, we investigated the value of self-learning and AI-CAD assistance in inexperienced readers.

## Materials and methods

This study was approved by the Institutional Review Board of Severance Hospital and informed consent was obtained from all participants (No. 4–2022-1562).

### Study design

Between February and August 2023, we recruited 26 inexperienced readers (less than 1 year of experience in thyroid US) from 6 hospitals. These participants were medical residents or fellows specializing in various departments including radiology, internal medicine, surgery, and family medicine. At first, readers were asked to watch a 5-minute online lecture (available via https://youtu.be/pnF5vUaIovI, Korean only) on K-TIRADS (Korean Thyroid Imaging Reporting and Data System classification (19) and perform a pretest consisting of 120 US images to make binary decisions (benign vs malignant) and assess K-TIRADS categories. Next, readers learned with a training set of 3,000 US images using an online platform, designed to consecutively display single nodule images, each accompanied by a binary diagnosis of benign or malignant. The platform allowed readers to adjust the playback speed according to their preferences. After completing the learning session, readers immediately repeated the same test as the pretest. Lastly, they underwent the test again, this time with AI assistance, using the SERA (SEveRance Artificial intelligence) program described in the following section. They were asked to complete training and testing within two weeks, and while the pace of online learning was adjusted to each individual, the readers had to record the time taken to study all 3,000 cases and the time spent on testing (**Table 1**).

**Table 1:** General information on the 26 inexperienced readers from 6 hospitals.

	Total (%)	Radiology department (n=18)	Other departments (n=8)	p-value
Department Radiology Internal medicine Surgery Family medicine	18 (69.2) 2 (7.7) 2 (7.7) 4 (15.4)			
Duration of previous thyroid US (month, SD)	2.3 ± 3.0	2.4 ± 2.6	2.4 ± 4.1	0.969
Time required for self-learning (min, SD)	222 ± 120	247 ± 140	165 ± 42	0.122
Time required for test with AI assistance (min, SD)	83 ± 59	83 ± 68	83 ± 41	0.990

Min, minute; SD, standard deviation; AI, artificial intelligence.

### Learning and test sets

We selected 3,000 images from 13,560 image sets utilized in a previous study (13). Images that demonstrated the most significant mean accuracy enhancement compared to earlier data points were selected, and these images made up Set 3 in the preceding study (13). The mean age of patients from whom the US images were derived for the learning set was 48.2 ± 13.8 years, and 81% of the patients were women. The mean size of the nodules was 20.0 ± 11.0 mm, with 49% being benign and 51% malignant, the latter of which 98.8% were identified as papillary thyroid carcinoma.

The test set, which was not included in the learning set, included 120 surgically confirmed thyroid nodules. The sample size for the test set was determined through estimations of the effect size, non-centrality parameters, denominator degrees of freedom, and power calculations. The mean age of patients from whom the US images were obtained for the test set was 43.7 ± 12.4 years, and 78.3% of the patients were women. The mean size of the nodules was 20.1 ± 9.4 mm. In terms of pathology, 48% of the nodules were benign and 52% were malignant, with a vast majority (93.5%) of the malignant nodules being classified as papillary thyroid carcinoma.

The standard reference of the test set for K-TIRADS assessment was consensus among the three experienced readers (5, 13, 23 years of experience in thyroid imaging). For reference, their intraclass correlation coefficient (ICC) was 0.908 (95% CI 0.876–0.933).

### AI-CAD application

**Figure 1:**
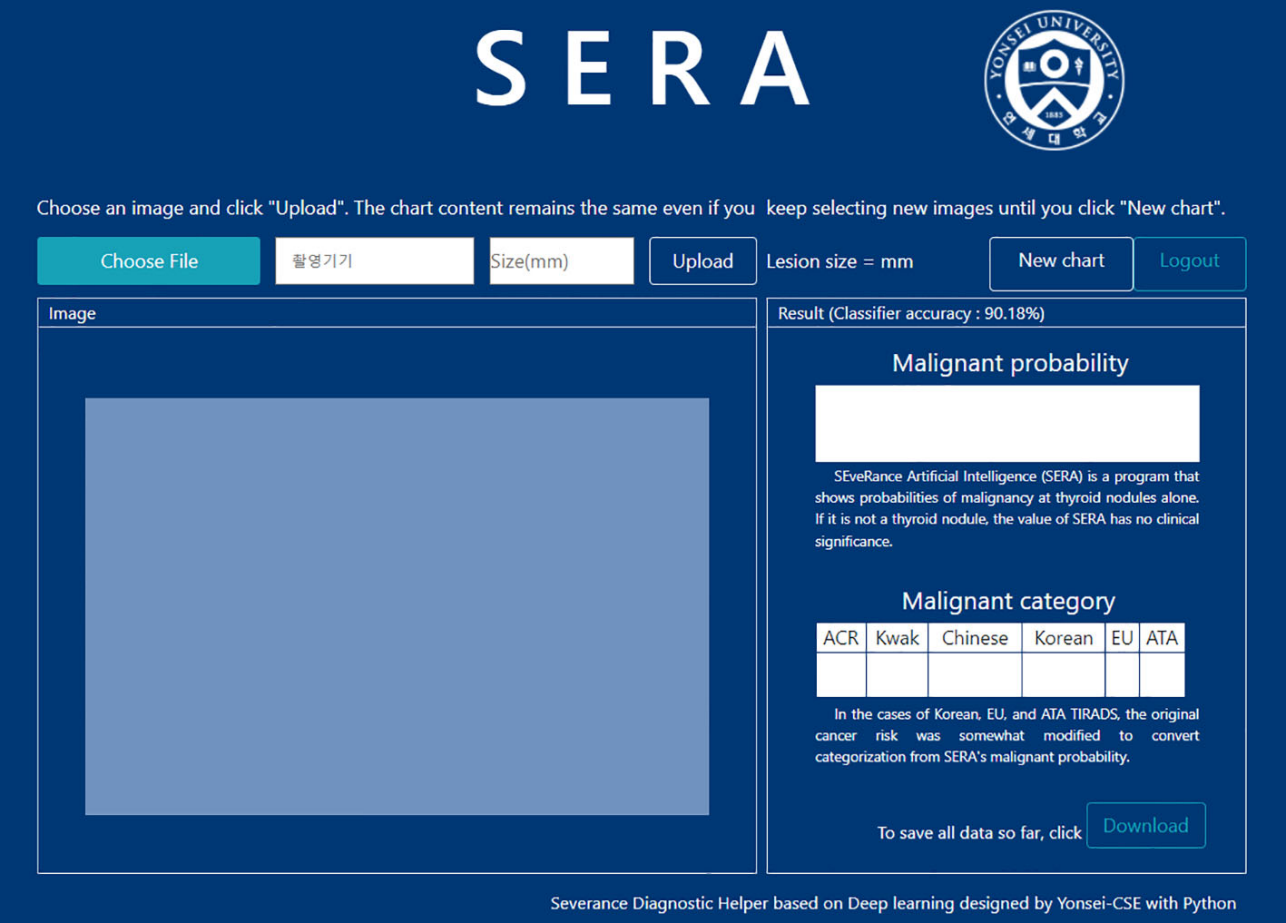
Image of the working process of the SERA program. When an US image is uploaded and cropped by the user, SERA presents the binary result (benign or malignant) with a malignant probability score. SERA, SEveRance Artificial intelligence program.

SERA is an online deep learning-based computer-aided diagnosis program trained with 13,560 US images of thyroid nodules that were surgically confirmed or cytologically proven as benign (category II) or malignant (category VI) on the Bethesda system and larger than 1cm in size (12). When users upload an US image cropped around the focal thyroid lesion according to user preference, SERA provides continuous numbers between 0 and 100, which correspond to the probability of the given test image being malignant (**Figure 1**). Since SERA presents results that are dependent on how images are cropped and which images are uploaded, the SERA scores are impacted by the initial judgments of users. In prior research, SERA showed comparable diagnostic performance to expert radiologists in an external validation set for diagnosing thyroid nodules (12).

### Statistical analysis

Sensitivity, specificity, accuracy and area under the receiver operating characteristic curve (AUC) were used to assess the diagnostic performance of each inexperienced reader. Interobserver agreement was quantified by the ICC. A two-sample t-test was used to detect differences between groups, specifically readers of radiology against readers of other specialties. The paired t-test was used to assess changes in diagnostic performance within the same group throughout the training program.

All statistical analyses were performed using SPSS (version 26.0) and MedCalc 22.009 (MedCalc Software, Oostende, Belgium). A p-value of 0.05 or less was considered statistically significant.

## Results

Among 26 participants, 18 readers were radiology residents (1^st^ and 2^nd^ year), and the other 8 were 4 fellows in endocrinology and surgery and 4 residents in family medicine (3^rd^ year). All 26 readers had none to little experience with thyroid US (range 0–10 months). The learning process for the 3,000 sets took an average of 222 minutes, and the test for the 120 sets utilizing AI assistance was completed in an average of 85 minutes (**Table 1**). There was no statistical difference in the duration of exposure between radiology residents and readers of other specialties (**Table 1**).

### Changes in diagnostic performance after self-learning

After self-learning with 3,000 cases, 26 readers improved accuracy (68.0% vs 71.2%, p=0.037) and AUC (0.679 vs 0.713, p=0.027) compared to their pretest performance (**Table 2**).

**Table 2:** Changes in the mean diagnostic performance of 26 readers during the learning program.

	Pretest	Posttest^*^	P-value^†^	AI-assistance	P-value^‡^
Sensitivity (%)	70.2 ± 15.7	69.6 ± 13.4	0.857	77.2 ± 8.7	0.002
Specificity (%)	65.7 ± 24.7	73.1 ± 20.0	0.126	74.3 ± 18.0	0.584
Accuracy (%)	68.0 ± 6.6	71.2 ± 6.4	0.037	75.8 ± 5.1	0.001
AUC	0.679 ± 0.07	0.713 ± 0.07	0.027	0.758 ± 0.06	0.001
ICC	0.575 ± 0.12	0.601 ± 0.13	0.104	0.590 ± 0.12	0.608

AI, artificial intelligence.

AUC, area under the receiver operating characteristic curve; ICC, intraclass correlation coefficients.

^*^ after self-learning.

^†^ Comparison between pretest and posttest.

^‡^ Comparison between posttest and test with AI assistance.

**Figure 2:**
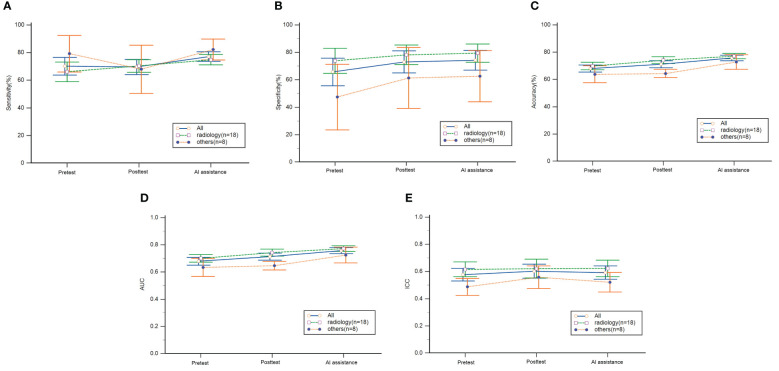
Mean diagnostic performance of readers during the learning program. **(A)** sensitivity, **(B)** specificity, **(C)** accuracy, **(D)** AUC and **(E)** ICC with 95% confidence intervals. The pretest was performed before self-learning and the posttest was performed after self-learning. AI, artificial intelligence; AUC, area under the receiver operating characteristic, ICC, intraclass correlation coefficients.

We separated 18 readers of radiology residency from the remaining 8 readers, and the pretest results of the radiology residents showed higher specificity (73.8% vs 47.4%, p=0.04) (**Table 3**). After self-learning, the radiology residents improved accuracy (69.9% to 74.2%, p=0.013) and AUC (0.7 to 0.743, p=0.016), but readers of other departments did not. Also, radiology residents showed better accuracy (74.2% vs 64.4%, p<0.001) and AUC (0.743 vs 0.647, p<0.001) than readers from other departments (**Table 3**, **Figure 2**).

**Table 3:** Changes in the mean diagnostic performance of 26 readers during the learning program compared between radiology residents and readers of other specialties.

	Pretest	Posttest^*^	P-value^†^	AI-assistance	P-value^‡^
Sensitivity (%)					
Radiology	66.1 ± 14.4	70.3 ± 9.1	0.125	74.9 ± 7.8	0.023
Other	79.2 ± 18.3	67.9 ± 20.9	0.145	82.3 ± 9.0	0.024
P-value^§^	0.067	0.763		0.069	
Specificity (%)					
Radiology	73.9 ± 18.3	78.3 ± 14.4	0.323	79.5 ± 13.4	0.637
Other	47.4 ± 28.6	61.4 ± 26.5	0.273	62.7 ± 22.4	0.795
P-value^§^	0.04	0.129		0.08	
Accuracy (%)					
Radiology	69.9 ± 5.4	74.2 ± 4.9	0.013	77.1 ± 3.9	0.046
Other	63.9 ± 7.4	64.4 ± 3.7	0.862	72.8 ± 6.5	0.007
P-value^§^	0.066	<0.001		0.115	
AUC					
Radiology	0.7 ± 0.06	0.743 ± 0.51	0.016	0.772 ± 0.04	0.053
Other	0.633 ± 0.08	0.647 ± 0.38	0.671	0.725 ± 0.07	0.006
P-value^§^	0.059	<0.001		0.111	
ICC					
Radiology	0.615 ± 0.11	0.621 ± 0.14	0.771		
Other	0.485 ± 0.07	0.557 ± 0.1	0.008		
P-value^§^	0.002	0.203			

AI, artificial intelligence.

AUC, area under the receiver operating characteristic curve; ICC, intraclass correlation coefficients.

^*^ after self-learning.

^†^ Comparison between pretest vs. posttest.

^‡^ Comparison between posttest vs. with AI assistance.

^§^ Comparison between residents in radiology vs. readers of other specialties.

### Changes in diagnostic performance with AI assistance

For all readers, diagnostic performance improved more with AI assistance compared to posttest; sensitivity (69.6% vs 77.2%, p=0.002), accuracy (71.2% vs 75.8%, p=0.001) and AUC (0.713 vs 0.758, p=0.001) all improved (**Table 2**). In the radiology group, sensitivity increased from 70.3% to 74.9% (p=0.023), and accuracy from 74.2% to 77.1% (p=0.046). In the other departments group, sensitivity increased from 67.9% to 82.3% (p=0.024), accuracy from 62.4% to 72.8% (p=0.007), and AUC from 0.647 to 0.725 (p=0.006). Final sensitivity, specificity, accuracy and AUC were not statistically different between the two groups (**Table 3**, **Figure 2**).

### Changes in K-TIRADS assessment

When we calculated the ICC for K-TIRADS assessment in consensus with the three staff radiologists, the overall ICC for K-TIRADS assessment did not significantly change during self-learning (0.575 vs 0.601). In the subgroup analysis, the ICC of radiology residents was higher than the other department readers in the pretest (0.615 vs 0.485, p=0.002). However, the ICC of readers from other departments increased after self-learning, The ICC showed no statistical difference between the two groups after self-learning (0.621 vs 0.557, p=0.203) (**Table 3**). The ICC value for each reader before and after self-learning is shown in **Supplementary Table 1**.

## Discussion

In this study, we investigated the effectiveness of online-based self-learning for diagnosing thyroid cancer in 26 inexperienced readers from six different hospitals from diverse specialties. Furthermore, we examined the impact of AI assistance on their diagnostic performance for thyroid nodules. After training with a set of 3,000 images, both AUC and accuracy improved for all readers on average, and AI assistance further enhanced these metrics.

Previously, a similar method of self-learning was proposed with 13,560 images being learned by six college freshmen (13). The six freshmen also showed improved sensitivity, specificity, accuracy, and AUC. However, it took an average of 30 hours for these freshmen to learn with 13,560 images (13), and viewing 13,560 images at a specific learning location for this amount of time poses considerable challenges in real life. In this study, we provided 3,000 images and all training was executed via an online platform, enabling participants to learn in their personal space at their convenience and record their results subsequently. In our study, we trained individuals with little to no experience in thyroid US but found that those more likely to benefit from training were radiology residents, family medicine residents, endocrinology fellows, and surgery fellows. On average, our participants took a mean of 222 minutes to learn from the 3,000 images, and this training led to increase in accuracy and AUC.

When we performed a subgroup analysis according to the medical department, the benefit of digital self-learning was only significant in radiology residents. Although there was no statistical difference in the recorded duration of exposure in the learning session between the radiology and other department groups, radiology residents are continuously exposed to images and cases through lectures and conferences during their training. This aspect of learning is likely to differentiate them from readers from other medical specialties. For groups less familiar or exposed to US images or radiological diagnostics, self-learning with 3,000 images may simply not be enough to achieve significant increase in diagnostic accuracy. Given the variation in outcomes across different specialties, incorporating detailed explanations for correct or incorrect answers during the self-learning phase could potentially enhance understanding and retention, particularly for those less familiar with ultrasound imaging. This method could mirror more interactive learning approaches found in question banks, which have been shown to improve diagnostic skills by reinforcing learning points through immediate feedback.

After the self-learning process, the final test performance with AI-CAD assistance showed additional increases in sensitivity, AUC, and accuracy. Previous research has well-documented the increased advantage that AI-CAD offers to beginners in US (12, 20–24). AI-CAD appears to supplement self-learning by offering direct assistance on specific cases, rather than just amplifying the learning effect. Unlike digital self-learning, AI-CAD assistance was effective for all readers, regardless of whether they were from the radiology department or others.

Additionally, as K-TIRADS is predominantly used for image interpretation in Korea, we also sought to ascertain whether the self-learning program had an impact on K-TIRADS assessment. Although the overall ICC for K-TIRADS assessment did not improve with self-learning, the ICC of readers from other specialties increased to the ICC of radiology residents. While such categorical assessments are known to have high interobserver variability (25), if we take into consideration that our standard reference group of experienced readers had an ICC of 0.908, we can assume that K-TIRADS assessments by inexperienced readers need further calibration. The challenges of these assessments appear hard to overcome with image-diagnosis set training.

Our study was conducted entirely on an online platform, enabling participants to learn at their own pace and schedule. This approach facilitated the recruitment of participants from hospitals located in diverse regions. One major advantage of online learning is its ability to reduce the burden on instructors, offer flexibility in terms of time and location, and provide consistent education to a broad audience (26). The proliferation of online learning, especially post-COVID, means that learners today have a strong propensity for web- and social media-based curricula (27, 28). However, US education isn’t just about gaining knowledge; it encompasses the development of psychomotor skills, visual perception for image acquisition, interpretation, and integration into medical decision-making (29). While our online self-learning can address some of these aspects, we anticipate it being particularly effective as a preparatory step to enhance diagnostic performance and boost confidence before trainees handle real clinical situations.

Similarly, AI-based diagnostic augmentation has shown comparable trends in improving diagnostic performance across other medical fields such as dermatology, cardiology, and oncology, where it enhances accuracy and aids less experienced practitioners. The success of these applications suggests that the learning methods employed in our study could potentially be adapted to these fields. In line with expanding our understanding of AI’s utility in medical training, further research could involve testing readers of different experience levels, including senior radiology residents, fellows, and junior faculty. Such studies would help ascertain if even more senior readers can benefit from AI, potentially broadening the scope of AI tools in supporting ongoing professional development and decision-making processes across various stages of a medical career.

There are some limitations to our study. First, since our approach was entirely based on online learning and testing, we had limited control over the learning process. Although we restricted the learning period to two weeks, outcomes might differ between participants who studied intensively and those who learned sporadically. Second, we assessed the overall effects on 26 learners from various medical departments, but the standard deviation of performance due to their different specialty backgrounds was substantial, especially for readers from other specialties than radiology. This variability makes it challenging to achieve statistical significance. Third, we evaluated performance based on binary diagnoses, which may seem overly simplistic. Finally, although we provided a set of 3000 cases for the one-time self-learning session, repetitive training might change the results.

In conclusion, In conclusion, our study demonstrated that while AI-CAD assists all inexperienced readers in improving diagnostic performance for thyroid nodules, the effectiveness of self-learning appears more pronounced in radiology residents, likely due to their prior ultrasonography knowledge. Further studies could explore its impact on other non-radiologist groups.

## Data availability statement

The raw data supporting the conclusions of this article will be made available by the authors, without undue reservation.

## Ethics statement

The studies involving humans were approved by Ethical Review Board of Severance Hospital. The studies were conducted in accordance with the local legislation and institutional requirements. The participants provided their written informed consent to participate in this study.

## Author contributions

SL: Data curation, Methodology, Writing – original draft, Writing – review & editing. HK: Data curation, Methodology, Writing – review & editing. HJ: Data curation, Writing – review & editing. JJ: Data curation, Writing – review & editing. JJ: Data curation, Writing – review & editing. JL: Data curation, Writing – review & editing. HH: Methodology, Visualization, Writing – review & editing. EL: Formal analysis, Software, Writing – review & editing. DK: Formal analysis, Software, Writing – review & editing. JK: Conceptualization, Funding acquisition, Project administration, Supervision, Writing – original draft, Writing – review & editing.
