# Corrigendum: Improving the diagnostic performance of inexperienced readers for thyroid nodules through digital self-learning and artificial intelligence assistance

**DOI:** 10.3389/fendo.2024.1466012

**Published:** 2024-09-02

**Authors:** Si Eun Lee, Hye Jung Kim, Hae Kyoung Jung, Jin Hyang Jung, Jae-Han Jeon, Jin Hee Lee, Hanpyo Hong, Eun Jung Lee, Daham Kim, Jin Young Kwak

**Affiliations:** ^1^ Department of Radiology, Yongin Severance Hospital, College of Medicine, Yonsei University, Yongin-si, Republic of Korea; ^2^ Department of Radiology, Kyungpook National University Chilgok Hospital, Daegu, Republic of Korea; ^3^ Department of Radiology, CHA University Bundang Medical Center, Seongnam-si, Republic of Korea; ^4^ Department of Surgery, Kyungpook National University Chilgok Hospital, Daegu, Republic of Korea; ^5^ Department of Endocrinology, Kyungpook National University Chilgok Hospital, Daegu, Republic of Korea; ^6^ Department of Radiology, Keimyung University Dongsan Hospital, Daegu, Republic of Korea; ^7^ Department of Computational Science and Engineering, Yonsei University, Seoul, Republic of Korea; ^8^ Department of Endocrinology, College of Medicine, Yonsei University, Seoul, Republic of Korea; ^9^ Department of Radiology, College of Medicine, Yonsei University, Seoul, Republic of Korea

**Keywords:** thyroid cancer, artificial intelligence, ultrasound, learning, digital learning

In the published article, an author name was incorrectly written as Jing Hyang Jung. The correct spelling is Jin Hyang Jung.

The authors apologize for this error and state that this does not change the scientific conclusions of the article in any way. The original article has been updated.

