# Supplementary material for: Improving the diagnostic performance of inexperienced readers for thyroid nodules through digital self-learning and artificial intelligence assistance
Source: Front Endocrinol (Lausanne). 2024 Jul 2;15:1372397. doi: 10.3389/fendo.2024.1372397 (PMC11249553; doi:10.3389/fendo.2024.1372397)
Supplement: Supplementary file 1 [file Table_1.docx]

Supplementary Table 1. Intraclass correlation coefficients of readers before and after self-learning.

|  |  | Pretest | Posttest^*^ | P-value |
| --- | --- | --- | --- | --- |
| Radiology | R1 | 0.485 | 0.330 |  |
|  | R2 | 0.361 | 0.778 |  |
|  | R3 | 0.526 | 0.752 |  |
|  | R4 | 0.566 | 0.686 |  |
|  | R5 | 0.523 | 0.688 |  |
|  | R6 | 0.752 | 0.601 |  |
|  | R7 | 0.413 | 0.603 |  |
|  | R8 | 0.551 | 0.516 |  |
|  | R9 | 0.464 | 0.639 |  |
|  | R10 | 0.656 | 0.700 |  |
|  | R11 | 0.459 | 0.672 |  |
|  | R12 | 0.450 | 0.585 |  |
|  | R13 | 0.683 | 0.728 |  |
|  | R14 | 0.672 | 0.701 |  |
|  | R15 | 0.577 | 0.613 |  |
|  | R16 | 0.682 | 0.682 |  |
|  | R17 | 0.646 | 0.686 |  |
|  | R18 | 0.580 | 0.701 |  |
|  | Average | 0.615 | 0.621 | 0.771 |
| Other departments | R1 | 0.582 | 0.582 |  |
|  | R2 | 0.075 | 0.380 |  |
|  | R3 | 0.602 | 0.652 |  |
|  | R4 | 0.137 | 0.551 |  |
|  | R5 | 0.484 | 0.435 |  |
|  | R6 | 0.320 | 0.349 |  |
|  | R7 | 0.387 | 0.495 |  |
|  | R8 | 0.618 | 0.428 |  |
|  | Average | 0.485 | 0.557 | 0.008 |
| Overall |  | 0.575 | 0.601 | 0.104 |

^*^after self-learning.
